# Patterns of therapy initiation during the first decade for patients with follicular lymphoma who were observed at diagnosis in the rituximab era

**DOI:** 10.1038/s41408-021-00525-0

**Published:** 2021-07-17

**Authors:** Arushi Khurana, Raphael Mwangi, Stephen M. Ansell, Thomas M. Habermann, James R. Cerhan, Christopher Strouse, Brian K. Link, Yucai Wang, Rebecca L. King, William R. Macon, J. C. Villasboas, Thomas E. Witzig, Matthew J. Maurer, Grzegorz S. Nowakowski

**Affiliations:** 1 Division of Hematology, Mayo Clinic, Rochester, MN USA; 2 Department of Quantitative Health Sciences, Mayo Clinic, Rochester, MN USA; 3 Department of Medicine, University of Iowa, Iowa City, IA USA; 4 Department of Laboratory Medicine and Pathology, Mayo Clinic, Rochester, MN USA

**Keywords:** Disease-free survival, B-cell lymphoma

## Abstract

Immediate treatment for asymptomatic, low-tumor burden follicular lymphoma (FL) has not shown an overall survival benefit over “watch and wait” (W/W) strategy. We estimated incidence of treatment initiation at specific time points and assessed its association with the presence of any criteria such as GELF, BNLI, GITMO at diagnosis. FL patients managed by W/W strategy were identified from the Molecular Epidemiology Resource (MER) of the University of Iowa/Mayo Clinic Lymphoma SPORE between 2002 and 2015. Cumulative incidence estimates of treatment initiation were calculated using transformation (as the first event) and death as competing risks. 401 FL patients were identified on W/W strategy. At a median follow-up of 8 years, 256 (64%) initiated treatment. For patients on the W/W strategy for 5 years, the likelihood of treatment initiation in the next 5 years was 12% compared to 43% at diagnosis unlike transformation rates which remained steady. Patients with any of popular treatment criteria at diagnosis did not have increased therapy initiation rates (44% vs. 42%) during the first 5 years or lymphoma-related death rates at 10 years (6% vs. 7%). Identifying biological differences in patients with early vs. late or no progression is a critical next step in understanding outcomes in W/W patients.

## Introduction

Watch and wait (W/W) remains a viable option in the rituximab era for asymptomatic, stage II–IV, low-tumor burden follicular lymphoma (FL) patients [1–3]. Studies to-date both in the pre- and post-rituximab era have not shown an overall survival (OS) benefit from immediate treatment in such low-risk patients [1, 3–6]. There are caveats to the W/W strategy, such as ensuring the patient is asymptomatic, no critical organ function is compromised or at threat from compression effect, no cytopenias, and the patient understands the advantages and problems related to deferring treatment. In the pre-rituximab era, no OS benefit was seen between immediate versus deferred treatment, so the W/W strategy was preferred over chemotherapy due to avoidance of toxicities [7]. However, the option of rituximab monotherapy, which is highly efficacious and significantly less toxic than chemotherapy, poses a challenge to the W/W strategy [8, 9]. Also, rituximab monotherapy has been shown to prolong time to next treatment, time to next chemotherapy, alleviate anxiety, and decrease the risk of histologic transformation [2, 3, 10, 1, 11, 12]. Criteria such as Groupe d’Etude des Lymphomes Folliculaires (GELF), British National Lymphoma Investigation (BNLI), and Gruppo Italiano Trapianto Midollo Osseo (GITMO) help to identify patients with low-tumor burden disease [4, 5, 13]. Practicing clinicians and clinical trials incorporate these standardized criteria to identify patients for immediate treatment and facilitate a discussion regarding treatment strategies. W/W strategy can be implemented successfully only when it aligns with both the patient and treating physician’s preferences and values, as the concept of no treatment in the setting of an incurable malignancy with a long survival, such as FL, may generate considerable stress and anxiety.

To improve the understanding and to better counsel patients regarding W/W strategy, this analysis sought to estimate the incidence of treatment initiation in our prospectively observed cohort of patients with FL who were registered in the Molecular Epidemiology Resource (MER) of the University of Iowa/Mayo Clinic Lymphoma Specialized Program of Research Excellence (SPORE). We further evaluated the association between the presence of any treatment initiation criteria (GELF, BNLI, or GITMO) at diagnosis and patterns of treatment initiation, transformation rates, and cause of death in FL patients managed by W/W.

## Methods

From 2002 to 2015, consecutive patients with newly diagnosed FL (grades 1, 2, and 3a) were offered enrollment to the MER [14]. A written informed consent was obtained from all patients; respective institutional review boards approved the study at the University of Iowa and Mayo Clinic. Patients were managed in agreement with the treating physician’s choice and were followed prospectively. Baseline clinical and pathological data were abstracted using a standard protocol. All patients were systematically contacted every 6 months for the first 3 years and then annually thereafter. Events such as death and transformation were verified through a review of pathology and medical records. For the current analysis, the inclusion criteria were initial diagnosis of FL grade I–IIIa, stage II–IV, managed by W/W strategy at diagnosis. Patients were classified as W/W if the management plan indicated observation per the treating physician’s clinical notes. Also, patients in the MER who were not classified as W/W but initiated therapy beyond 6 months from diagnosis are standardly re-reviewed for initial treatment classification [6, 15]. Patients with composite diagnosis, FL grade 3b, and histological transformation at the time of diagnosis identified on pathology were excluded.

Patients were retrospectively considered to meet treatment criteria at diagnosis if they had any GELF, BNLI, or GITMO components per available abstracted data in the MER database. Components of GELF, BNLI, and GITMO criteria not available in MER were organ compression, effusions, life-threatening organ involvement, discomfort due to tumor masses, and rapid generalized disease progression in the last 3 months. Since not all criteria were prospectively assessed and captured for all patients, we use the term “treatment initiation criteria” for those meeting any of the available components of these three criteria and note that it will be conservative for formal GELF, BNLI, and GITMO assessment. Cause of death was obtained from death certificates and review of medical records. The specific cause was assigned in the MER by one of the study physicians per-protocol developed for the ECOG E4494 protocol [16]. The primary goal was to estimate the initiation of treatment for FL. Because more aggressive treatments are required for patients with the transformed disease, transformation before initiation therapy was analyzed as a competing risk. The estimates for treatment initiation and transformation were then combined for a total treatment event estimate.

OS was defined as the time from diagnosis date to the date of death from any cause. OS was analyzed using the Kaplan–Meier method. Time to treatment initiation was defined as the time from diagnosis until the initiation of first therapy. Cumulative incidence estimates of treatment initiation were calculated using transformation to diffuse large B-cell lymphoma (DLBCL), or high-grade B-cell lymphoma and death due to any cause as competing risks utilizing the cuminc function from the cmprsk package in R version 3.6.2 [17]. Cause of death was analyzed in a similar competing risks manner. Transformation was defined based on biopsy-proven disease. All analyses were performed using R version 3.6.2, and SAS version 9.4M5.

## Results

### Patient characteristics

A total of 401 patients with newly diagnosed FL grade 1–3a, stage II–IV were identified in the MER cohort for whom initial therapy was deferred, hence classified as W/W strategy. The baseline characteristics of the W/W patient cohort are shown in Table 1. The median age was 61 years (IQR 52–70), and 50% were females. The majority of patients with a W/W strategy had favorable clinical and prognostic factors such as normal LDH (89%), hemoglobin >12 gm/dL (91%), no B-symptoms (97%), and low-intermediate (0–2) FLIPI score (83%). At least one treatment initiation criterion was met in 54% (218/401) patients. At a median follow-up of 8 years (IQR 5.9–12), 256 (64%) patients had initiated treatment (including 32 with transformation before treatment), and there were 78 (19%) deaths

**Table 1:** Baseline characteristics of the follicular lymphoma patient cohort initiated on W/W strategy.

Characteristics	Total (*N* = 401)
Median age	61 (IQR 52.0–70.0)
Age >60	202 (50.4%)
Male	202 (50.4%)
Ann arbor stage III/IV	247 (62.4%)
ECOG performance status <2	386 (97.2%)
Absence of B symptoms	388 (97%)
LDH ≤ ULN	317 (88.8%)
FLIPI	
Low (0–1) Intermediate (2) High (≥3)	194 (48.4%) 139 (34.7%) 68 (16.9%)
Number of nodal groups >4	109 (27.6%)
Bone marrow involvement	139 (35.5%)
Met treatment initiation criteria	218 (54%)

### Patterns of treatment initiation, transformation, and death

**Fig. 1:**
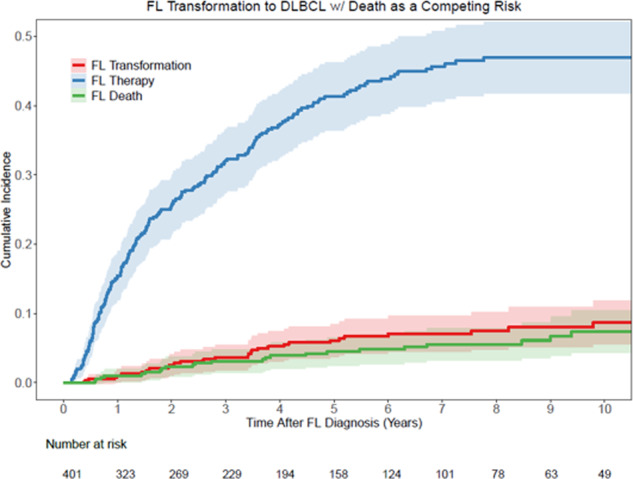
Estimates of treatment initiation from the time of diagnosis. Cumulative incidence estimates of treatment initiation with transformation and death as competing risks.

Cumulative incidence estimates of treatment initiation from diagnosis are shown in Fig. 1 and Table 2. At 2 years from diagnosis, the likelihood of treatment initiation was 26% (95% CI: 22–31), the incidence of untreated transformation was 3% (95% CI:1–5), and death without initiating treatment was 2% (95% CI: 1–4). At 10 years, this increased to 48% (95% CI: 43–54), 9% (95% CI:6–13), and 6% (95% CI: 4–10), respectively. The OS (including death after treatment initiation) of the W/W cohort was 73% (95% CI: 68–80) at 10 years. The cause of death was noted to be 7% (95% CI: 4–11) from progressive lymphoma, 11% (95% CI: 8–15) from non-lymphoma-related causes, and 9% (95% CI: 5–15) unknown causes.

**Table 2:** Cumulative incidence estimates of treatment initiation, transformation to DLBCL before treatment, and death without therapy based on the duration of W/W strategy.

Time from start of W/W	FL status	Likelihood (point estimates) in the next 1 year (95% CI)	Likelihood (point estimates) in the next 2 years (95% CI)	Likelihood (point estimates) in the next 5 years (95% CI)	Likelihood (point estimates) in the next 10 years (95% CI)
Diagnosis	Transformation prior to therapy	0.01 (0.00, 0.03)	0.03 (0.01, 0.05)	0.06 (0.04, 0.09)	0.09 (0.06, 0.13)
	Initiation of therapy	0.16 (0.13, 0.20)	0.26 (0.22, 0.31)	0.43 (0.38, 0.48)	0.48 (0.43, 0.54)
	Death without therapy	0.01 (0.00, 0.03)	0.02 (0.01, 0.04)	0.04 (0.03, 0.07)	0.06 (0.04, 0.10)
2 years	Transformation	0.02 (0.01, 0.04)	0.04 (0.02, 0.07)	0.06 (0.04, 0.10)	0.09 (0.06, 0.14)
	Therapy	0.10 (0.07, 0.14)	0.18 (0.14, 0.23)	0.30 (0.25, 0.37)	0.39 (0.32, 0.48)
	Death	0.01 (0.00, 0.03)	0.02 (0.01, 0.05)	0.04 (0.02, 0.07)	0.10 (0.06, 0.19)
4 years	Transformation	0.02 (0.01, 0.05)	0.03 (0.01, 0.07)	0.05 (0.03, 0.11)	0.09 (0.04, 0.19)
	Therapy	0.08 (0.05, 0.14)	0.13 (0.09, 0.19)	0.19 (0.14, 0.26)	0.36 (0.26, 0.50)
	Death	0.01 (0.00, 0.04)	0.02 (0.01, 0.05)	0.02 (0.02, 0.06)	0.17 (0.09, 0.33)
5 years	Transformation	0.01 (0.00, 0.05)	0.02 (0.01, 0.06)	0.06 (0.02, 0.13)	0.08 (0.04, 0.20)
	Therapy	0.05 (0.03, 0.10)	0.09 (0.05, 0.16)	0.12 (0.07, 0.19)	0.31 (0.20, 0.47)
	Death	0.01 (0.00, 0.05)	0.01 (0.00, 0.06)	0.04 (0.02, 0.11)	0.18 (0.09, 0.37)
6 years	Transformation	0.01 (0.00, 0.06)	0.02 (0.00, 0.07)	0.05 (0.02, 0.12)	0.08 (0.03, 0.21)
	Therapy	0.04 (0.02, 0.10)	0.07 (0.04, 0.14)	0.13 (0.07, 0.23)	0.28 (0.17, 0.45)
	Death	0.02 (0.00, 0.06)	0.02 (0.00, 0.05)	0.06 (0.02, 0.16)	0.19 (0.09, 0.39)

The incidence of treatment initiation for FL plateaued over time as patients remained treatment-free after diagnosis. For patients on the W/W strategy successfully for 5 years without any treatment, the likelihood of treatment initiation in the next 5 years was 12% compared to 43% at diagnosis. Unlike the treatment initiation rates, the transformation rates in patients continuing on the W/W strategy remained steady. The likelihood of transformation in the next 5 years was 6% from diagnosis and after staying treatment-free for 5 years.

**Fig. 2:**
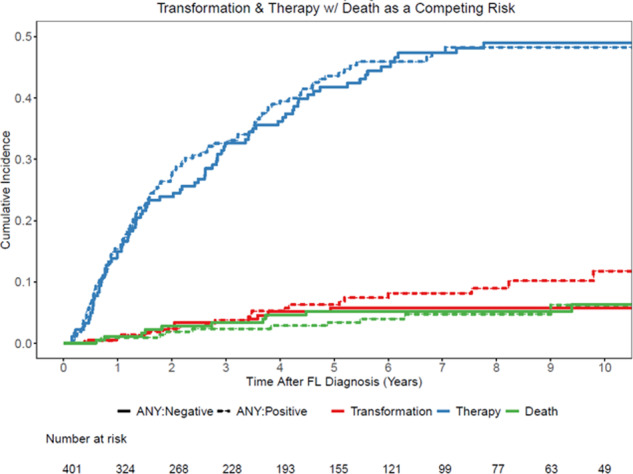
Estimates of treatment initiation, transformation and death based on treatment initiation criteria. Cumulative incidence estimates of treatment initiation, transformation and death based on presence or absence of any treatment initiation criteria.

**Fig. 3:**
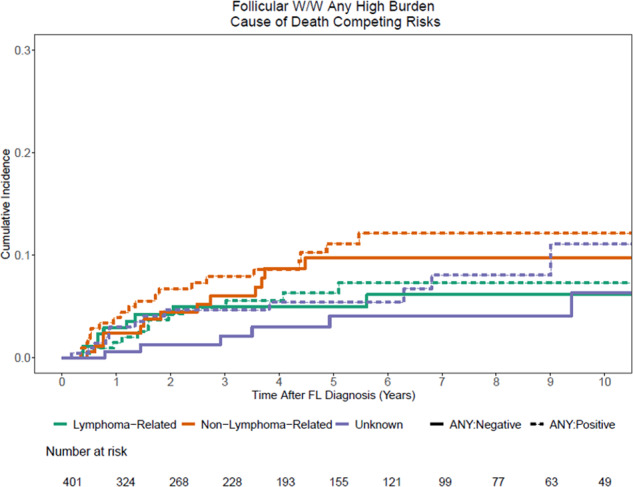
Cause of death estimates. Cumulative incidence estimates of cause of death based on presence of any treatment initiation criteria.

Two hundred and eighteen patients (54%) met at least one of the retrospectively applied treatment initiation criteria at diagnosis in this W/W cohort. These criteria were combined based on the components of GELF, BNLI, and GITMO criteria available in MER (Table 3). Patients that met at least one of the treatment initiation criteria at diagnosis did not have an increased rate of therapy initiation for FL (44% vs. 42%) or histologic transformation to DLBCL/HGBCL (6% vs. 6%) during the first 5 years from diagnosis (Table 4 and Fig. 2). Additionally, the lymphoma-related death rates were similar between the two groups (6% vs. 7%) at 10 years (Fig. 3). Similar analyses for treatment initiation, histologic transformation, and death based on individual criteria are provided in the Supplementary Figs. 1–3.

**Table 3:** Derivation of treatment initiation criteria and components of GELF, BNLI, and GITMO criteria available in MER database.

Any treatment initiation criteria (applied for the analysis)	GELF criteria	GITMO criteria	BNLI criteria
High tumor burden defined by —A tumor >10 cm (1.5%, *n* = 6, missing = 5), or >2 nodes in 3 distinct areas each >3 cm (2.7%, *n* = 11, missing = 19)	High tumor burden defined by —A tumor >7 cm,^b^ or >2 nodes in 3 distinct areas each > 3 cm^a^	Extra nodal disease^a^	Rapid generalized disease progression in the last 3 months
Symptomatic splenic enlargement (4.5%, *n* = 18, missing = 3)	Symptomatic splenic enlargement^a^	Spleen enlargement^a^	Life-threatening organ involvement
Presence of systemic symptoms (ECOG PS > 1) (2.8%, *n* = 11, missing = 4)	Organ compression	Leukemia phase^a^	Renal or macroscopic liver infiltration^a^ (4.9%, *n* = 20, missing = 0)
Serum LDH > ULN (10%, *n* = 40, missing = 44)	Ascites or pleural effusion	Serous effusions	Bone lesions^a^ (2.0%, *n* = 8, missing = 0)
Β2-microglobulin > ULN (21%, *n* = 85, missing = 261)	Presence of systemic symptoms (ECOG PS > 1)^a^	Nodal or extranodal mass >7 cm^b^	Presence of systemic symptoms^a^
Extra nodal disease (27%, *n* = 110, missing = 0)	Serum LDH > ULN^a^	>2 nodal masses, each >3 cm^a^	Hemoglobin <10 g/dL^a^
Leukemia phase (4.7%, *n* = 19, missing = 0)	Β2-microglobulin > ULN^a^	B symptoms^a^	WBC < 3.0 × 10^9^ related to marrow infiltration^a^
B symptoms (3.0%, *n* = 12, missing = 1)		Presence of systemic symptoms (ECOG PS > 1)^a^	
Cytopenia due to marrow infiltration; Hemoglobin < 10 g/dL; WBC < 3.0 × 10^9^ related to marrow infiltration (1.0%, *n* = 4, missing = 0)		Serum LDH or Β2-microglobulin > ULN^a^	
		ESR > ULN	
		Cytopenia due to marrow infiltration^a^	
Bone lesions (2.0%, *n* = 8, missing = 0)			

*PS* performance status, *LDH* lactate dehydrogenase, *ULN* upper limit of normal, *ESR* erythrocyte sedimentation rate, *WBC* white blood cell count.

^a^Captured in MER as described in the criteria

^b^Captured in MER with different cut off value, nodal mass >10 cm instead of 7 cm.

**Table 4:** Cumulative incidence estimates of treatment initiation, transformation to DLBCL before treatment, and death without therapy based on presence or absence of any treatment initiation criteria at diagnosis.

Time from start of W/W	FL status	Likelihood (point estimates) in the next 1 year (95% CI)	Likelihood (point estimates) in the next 2 year (95% CI)	Likelihood (point estimates) in the next 5 year (95% CI)	Likelihood (point estimates) in the next 10 year (95% CI)
Total cohort	Transformation	0.01 (0.00, 0.03)	0.03 (0.01, 0.05)	0.06 (0.04, 0.09)	0.09 (0.06, 0.13)
	Therapy	0.16 (0.13, 0.20)	0.26 (0.22, 0.31)	0.43 (0.38, 0.48)	0.48 (0.43, 0.54)
	Death	0.01 (0.00, 0.03)	0.02 (0.01, 0.04)	0.04 (0.03, 0.07)	0.06 (0.04, 0.10)
Any treatment initiation criteria: positive	Transformation	0.01 (0.00, 0.04)	0.02 (0.01, 0.06)	0.06 (0.04, 0.11)	0.12 (0.07, 0.19)
	Therapy	0.15 (0.11, 0.21)	0.27 (0.22, 0.34)	0.44 (0.37, 0.51)	0.48 (0.42, 0.56)
	Death	0.01 (0.00, 0.04)	0.02 (0.01, 0.05)	0.03 (0.02, 0.07)	0.06 (0.03, 0.12)
Any treatment initiation criteria: negative	Transformation	0.01 (0.00, 0.04)	0.03 (0.01, 0.07)	0.06 (0.03, 0.11)	0.06 (0.03, 0.11)
	Therapy	0.15 (0.11, 0.21)	0.24 (0.18, 0.31)	0.42 (0.35, 0.50)	0.49 (0.42, 0.57)
	Death	0.01 (0.00, 0.04)	0.03 (0.01, 0.07)	0.05 (0.03, 0.10)	0.06 (0.03, 0.12)

## Discussion

This report provides specific time point estimates (1, 2, 5, and 10 years) of treatment initiation based on the duration of W/W strategy in patients with FL managed in the rituximab era, which has not been reported to date. We also noted that the incidence of treatment initiation decreased over time as patients remained treatment-free after diagnosis. For patients on W/W strategy successfully for 5 years without any treatment, the likelihood of treatment initiation was 5%, 9%, and 12% in the next 1, 2, and 5 years, respectively as compared to 16%, 26%, and 43% at the start of W/W. This finding highlights the heterogeneous nature of FL, as the patients with a longer duration of W/W likely have more indolent biology, although studies to date have not adequately defined this population. This reinforces the notion that the odds of remaining on W/W increase with the W/W strategy’s increasing duration. It supports maintaining this strategy but also suggests the need to continue to follow these patients carefully.

Current literature suggests that rituximab monotherapy has remarkable response rates, delays the time to next treatment, including chemotherapy, has low toxicity, and psychological benefits [1–3, 11, 18]. However, there is not an OS benefit over the W/W strategy. The argument in favor of initial treatment of such low-tumor burden patients is the potential of keeping the tumor bulk low, preventing genetic changes from accumulating, decreasing the incidence of transformation, and potentially allowing the normal immune surveillance mechanisms to control the disease. In contrast, some argue against the earlier introduction of rituximab for its potential to introduce clinical resistance [19, 20]. While in vitro studies show acquired resistance to rituximab with repeated exposure, the extent of rituximab exposure inducing resistance in the clinical setting and the frequency of such resistance remains unknown. One retrospective study reported up to 17% of patients with rituximab resistance (defined as progression during induction, maintenance, or within 6 months from the last dose) in FL grades 1–3a with the first exposure to rituximab [21]. Two recent studies have shown that the progression-free survival (PFS) decreased with increasing lines of therapy [18, 22]. Link et al. reported longest PFS after first-line therapy (median 6.6 years), which diminished with each subsequent line (2nd—median 1.5 years, 3rd—0.83, 4th—0.69, and 5th— 0.68 years) [22]. While most treatment initiation after initial W/W was due to progressive disease (75%), there was still a significant proportion (23%) of treatment initiations due to patient or physician preference noted in the National LymphoCare Study [2]. It is, therefore, imperative that due consideration be given to the benefits of delaying the need for next treatment/chemotherapy, patient’s preference, and comfort while understanding the risks of treatment-related toxicity. A new rationale to delay treatment for asymptomatic patients is the worldwide COVID-19 pandemic. A UK study showed that the individuals with hematological malignancies undergoing treatment are at a higher risk of severe complications when diagnosed with COVID-19 [23, 24]. Additionally, the American Society of Hematology provided COVID-19 resources for indolent lymphomas and advised to defer treatment and monitor closely in patients with a borderline indication for therapy such as those meeting GELF criteria but asymptomatic [25]. While this may be temporary until mass vaccination occurs, it deserves consideration in the current times.

Previous reports provide estimates of PFS, time to next treatment, time to chemotherapy, and OS in patients undergoing W/W strategy versus rituximab (R) monotherapy or R-chemotherapy [1, 2, 6]. In the National LymphoCare study of 1754 patients with FL lymphoma, 386 (22%) had a first-line management strategy of W/W. Nastoupil et al. reported in this study, with a median follow-up of 8.1 years, that 62% (238/386) had initiated a second management strategy, of which 75% (179/238) were due to progressive disease, 14% (34/238) due to other reason (physician decision), and 9% (22/238) due to patient preference [2]. Another study reported that 50% of W/W strategy patients initiated a second treatment at a median follow-up of 5 years [6]. Similar to our analysis, a Danish Lymphoma Registry study reported time point-based estimates but included both the cumulative incidence of lymphoma treatment and deaths together at 3, 5, and 10 years [3]. Batlevi et al. have also recently reported that 34% of their W/W cohort (*n* = 461) did not initiate treatment at a median follow-up of 8.3 years [18]. Our report is consistent with previously reported studies, with a similar estimate of about 64% of patients initiating treatment with a median follow-up of 8 years.

Patients with W/W as an initial treatment strategy had more favorable clinical factors such as normal LDH, better ECOG performance status, normal hemoglobin, and low-intermediate risk FLIPI score. This suggests that the patients who initiated on W/W strategy overall have a good prognosis, which is consistent with other reports [1–3, 5, 18]. The overall histological transformation rates in the immunochemotherapy era have been reported as 2% per year; however, this also includes transformations after the first therapy [3, 10, 18, 26]. The untreated transformation risk in this report was 6% at 5 years. Batlevi et al. recently showed a histological transformation rate of 15.3% at a median follow-up of 8.3 years. Only 19% (31/164) transformed before receiving any therapy, while the majority of the transformation (81%) occurred after first-line therapy [18]. In the present study, transformation, and death were used as competing risks in the estimation of treatment initiation to discriminate treatment initiation for FL vs. transformed disease as the clinical management differs for these two scenarios.

The randomized clinical trials that compared W/W to rituximab monotherapy used criteria such as GELF or BNLI to identify patients with a low-tumor burden and asymptomatic disease not in need of immediate treatment. In the real world, clinicians sometimes take into consideration these factors while contemplating a treatment strategy. Previous reports have identified risk factors such as >4 nodal group involvement and increased LDH at diagnosis to be associated with increased lymphoma treatment/lymphoma-related death [3, 6]. The presence of any treatment initiation criteria at diagnosis in our dataset did not identify patients with either increased treatment initiation rates, histological transformation rates, or patients at higher risk of lymphoma-related deaths in W/W patients. We emphasize, however, that this retrospective inquiry on association with treatment criteria does not reflect all patients at time of presentation, only those that clinicians ultimately chose for deferral of systemic treatment. This highlights the heterogeneous nature of FL, even within the W/W subset of patients. There is a need for consideration of other factors in addition to GELF/BNLI/GITMO, which are both clinical (FL international prognostic index) and include genetic/molecular predictive models such as m7-FLIPI for better identification of patients suitable for W/W [27].

The decision to pursue a W/W strategy and comply with it over time is challenging in the era of minimizing treatment delays. Treatment initiation in the absence of strong clinical indications is thus sought on the basis of patient and physician’s preference and comfort with deferring treatment. A previous study has shown some psychological benefits with rituximab treatment over the W/W strategy based on the Mental Adjustment to Cancer scale score and Illness Coping Style score [1, 28, 29], Otherwise, there were no significant differences in symptom burden or quality of life, survival, or rate of disease transformation. In the phase III RESORT trial, there were no differences in illness-related anxiety and health-related QoL between the treatment arms, although it did not include a W/W arm [30, 11]. These results suggest that progression by itself may not increase anxiety, especially if patients anticipate progression and understand both the natural history of the disease along with available treatment options. Patients’ difficulties in coping with a chronic cancer diagnosis could be improved by more frequent counseling and reassurance based on literature, palliative care for support and coping skills, and psychotherapy [29, 31]. This report provides additional data for initial treatment discussion and aid in counseling patients regarding the likelihood of treatment initiation based on the duration of W/W.

This report’s strengths include a prospectively observed cohort with second management strategy initiation rates comparable to previously reported data suggesting minimal bias. Our median follow-up of ~8 years is long enough to capture events such as second treatment initiation, transformation, and death. Additionally, we used competing risk analysis to model the cumulative incidence of treatment initiation. Another strength of this real-world analysis includes information on combined GELF/BNLI/GITMO-based treatment initiation criteria and their influence on the selection of W/W as the initial strategy. As the application of criteria such as GELF, GITMO and BLNI were retrospective, we only used the criteria that were objectively measured such as nodal size, splenic enlargement, cytopenias, LDH, beta-2 microglobulin, to avoid the element of subjectivity that exists while evaluating patient symptoms. These factors are reliable as much as if this was a prospective evaluation. This study’s limitations include lack of control over the treating physician’s and patient’s discretion for selecting W/W as an initial strategy, follow-up visit intervals, and frequency of scans. Therefore, its impact on the outcomes is unknown, but this variability may be more reflective of real-world practice. Also, not all GELF/BNLI/GITMO criteria were prospectively assessed and captured for all patients as mentioned in the methods and will be conservative for formal GELF/BNLI/GITMO assessment.

## Conclusions

The likelihood of treatment initiation plateaued with a prolonged duration of W/W, highlighting heterogeneity within this indolent biology. Additional factors other than those listed in GELF/BNLI/GITMO criteria are required to appropriately select patients suited from W/W and avoid overtreatment of FL patients. This report provides additional guidance for patient counseling that may help alleviate anxiety regarding the need for treatment and cope better with this chronic and incurable lymphoma diagnosis.

## Supplementary information

Supplemental material
