# Supplementary material for: Patterns of therapy initiation during the first decade for patients with follicular lymphoma who were observed at diagnosis in the rituximab era
Source: Blood Cancer J. 2021 Jul 17;11(7):133. doi: 10.1038/s41408-021-00525-0 (PMC8286048; doi:10.1038/s41408-021-00525-0)
Supplement: Supplementary file 1 — Supplemental material [file 41408_2021_525_MOESM1_ESM.pdf]

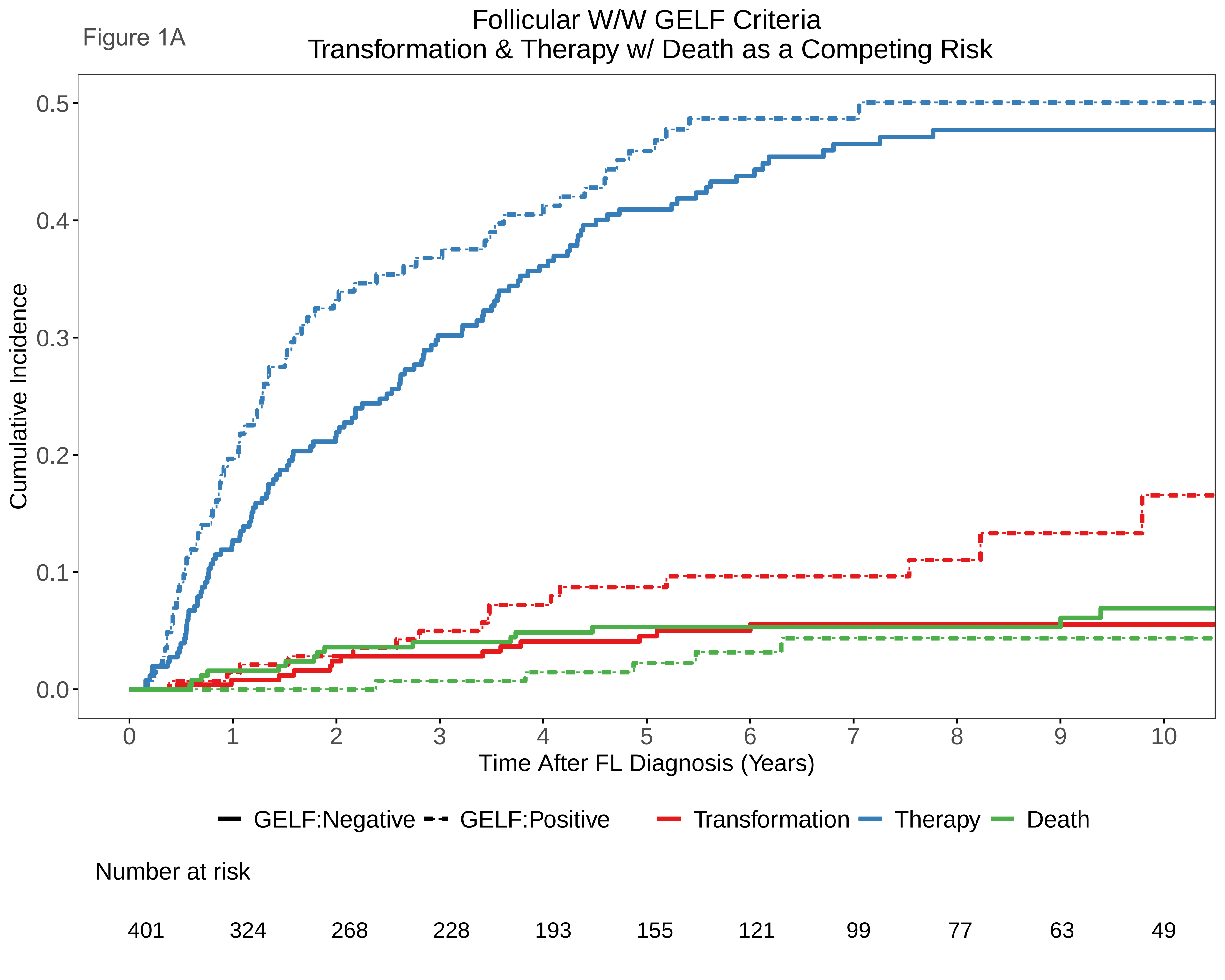
Figure 1A
Follicular W/W GELF Criteria
Transformation & Therapy w/ Death as a Competing Risk
Cumulative Incidence
0.0
0.1
0.2
0.3
0.4
0.5
0 1 2 3 4 5 6 7 8 9 10
Time After FL Diagnosis (Years)
GELF:Negative
GELF:Positive
Transformation
Therapy
Death
Number at risk
401 324 268 228 193 155 121 99 77 63 49

Figure 1B

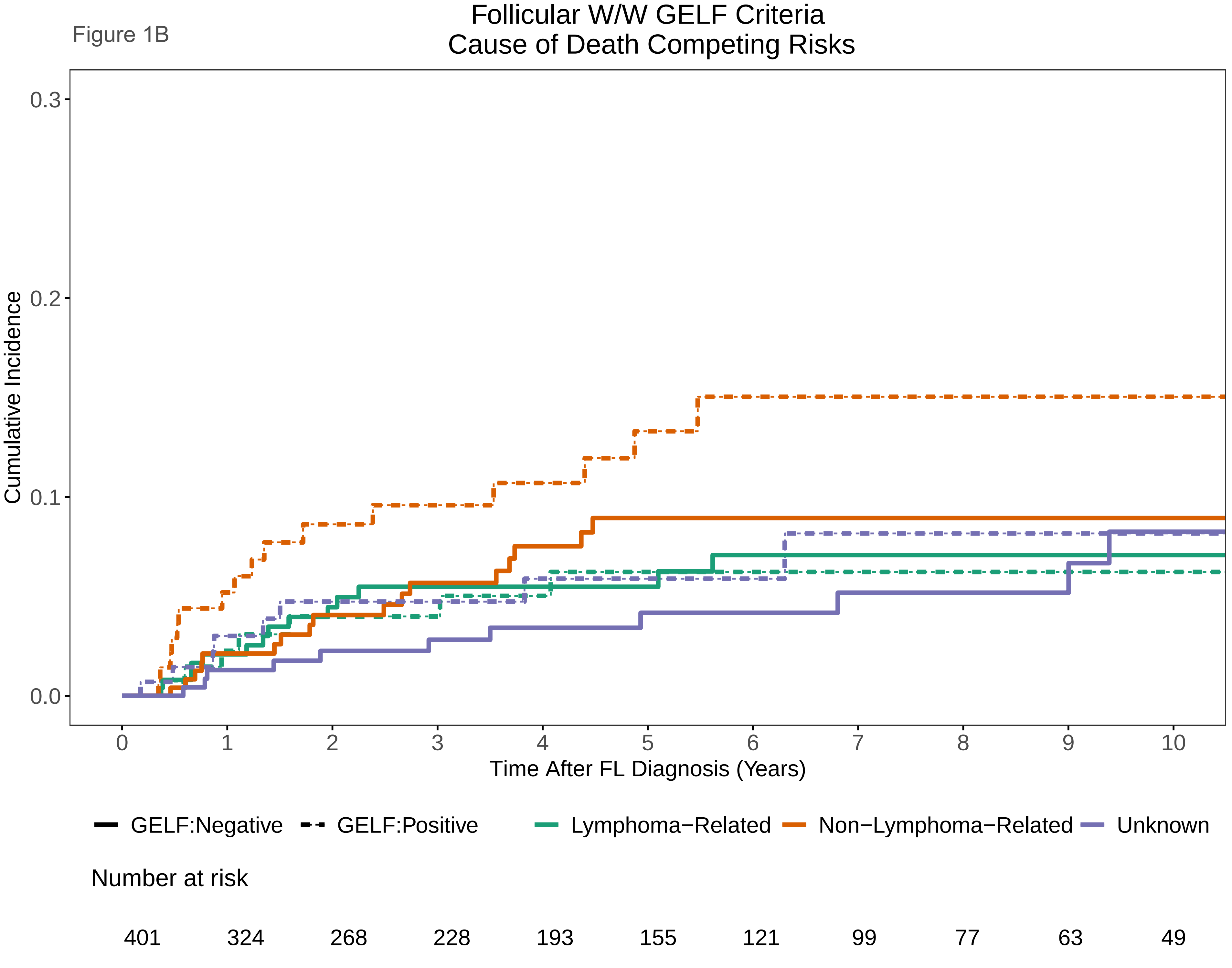
Follicular W/W GELF Criteria
Cause of Death Competing Risks
Cumulative Incidence
0.3
0.2
0.1
0.0
0
1
2
3
4
5
6
7
8
9
10
Time After FL Diagnosis (Years)
GELF:Negative
GELF:Positive
Lymphoma−Related
Non−Lymphoma−Related
Unknown
Number at risk
401
324
268
228
193
155
121
99
77
63
49

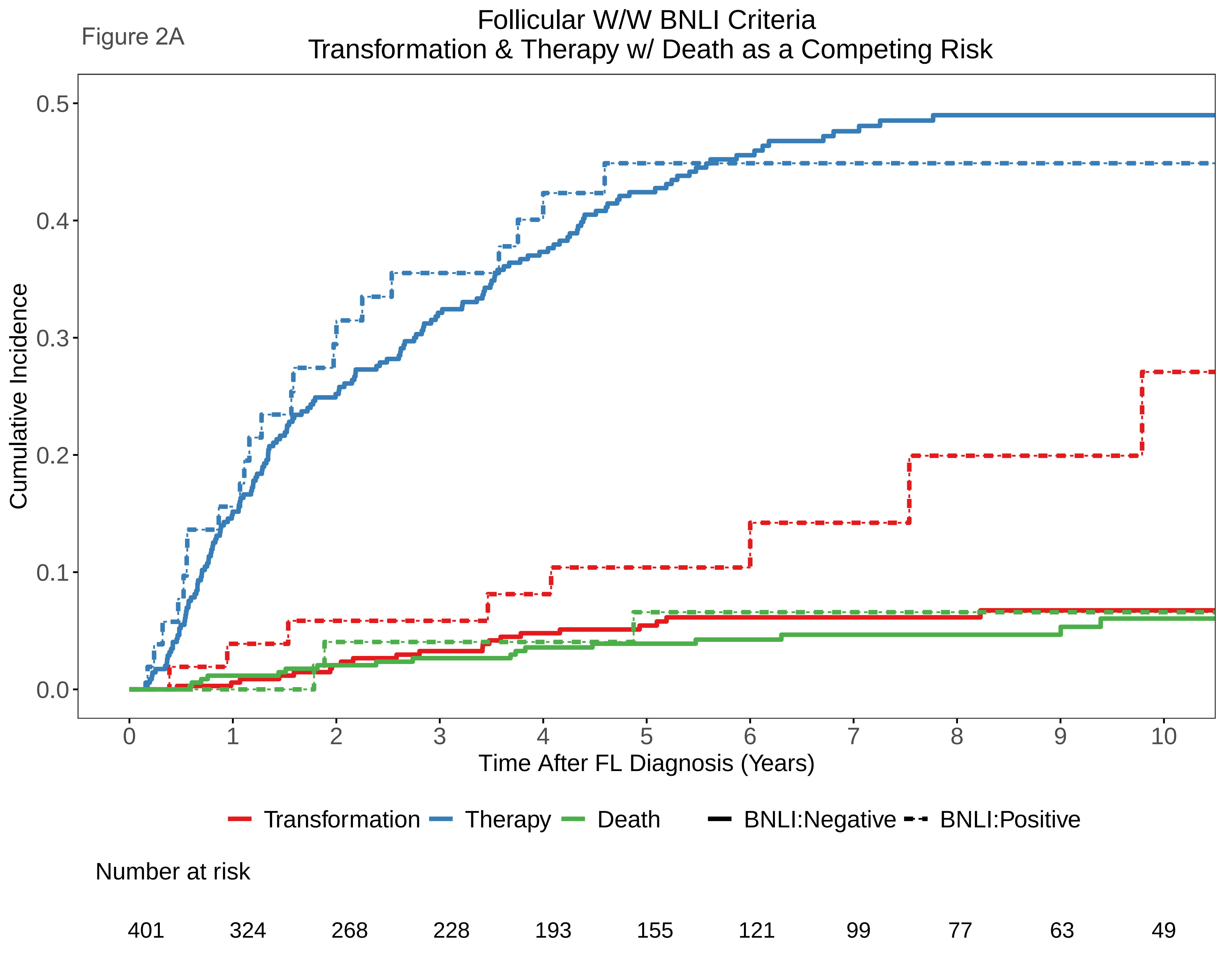
Figure 2A
Follicular W/W BNLI Criteria
Transformation & Therapy w/ Death as a Competing Risk
Cumulative Incidence
0.0
0.1
0.2
0.3
0.4
0.5
0
1
2
3
4
5
6
7
8
9
10
Time After FL Diagnosis (Years)
Transformation
Therapy
Death
BNLI:Negative
BNLI:Positive
Number at risk
401
324
268
228
193
155
121
99
77
63
49

Figure 2B

Follicular W/W BNLI Criteria
Cause of Death Competing Risks

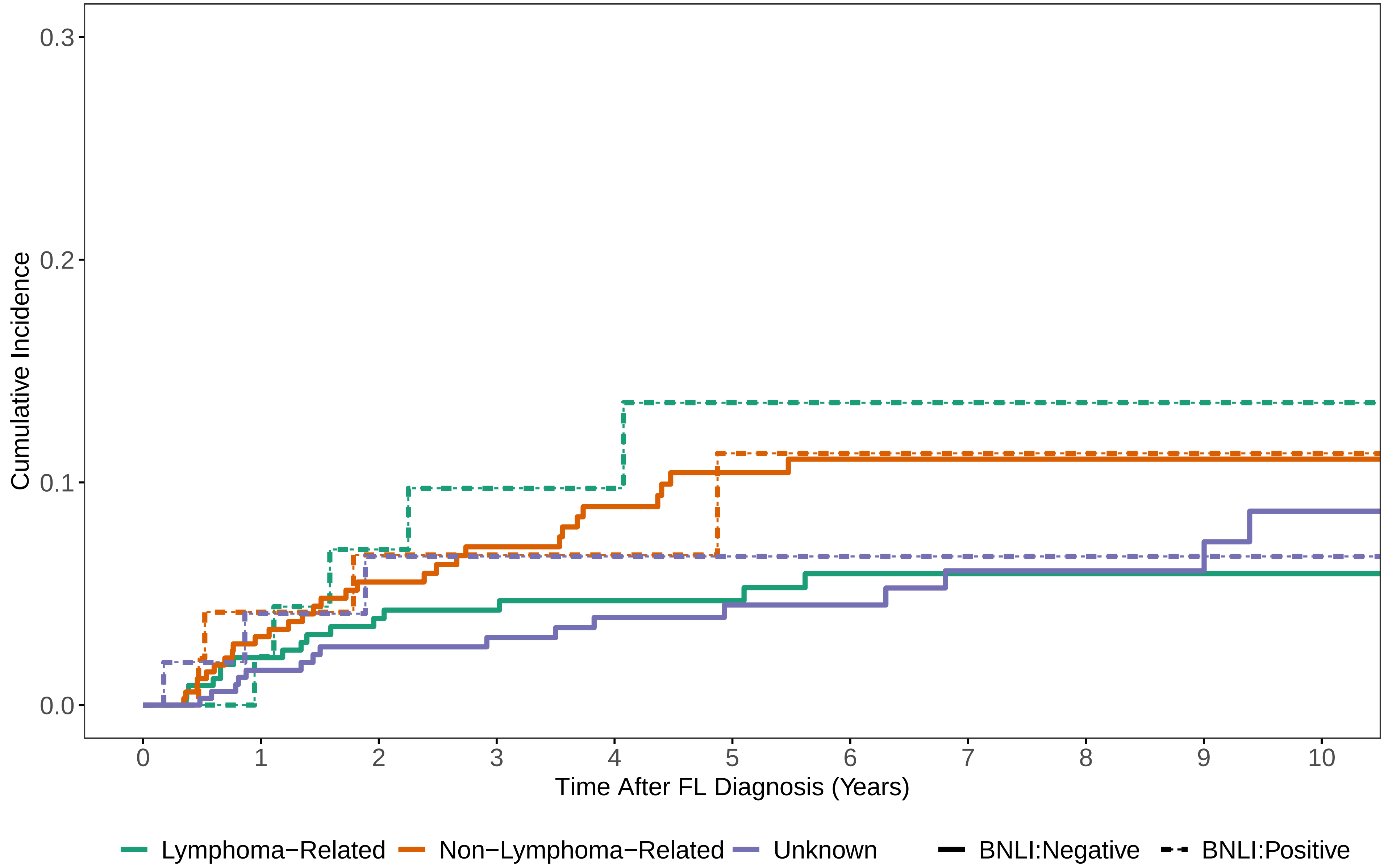

Number at risk

401	324	268	228	193	155	121	99	77	63	49

Figure 3A

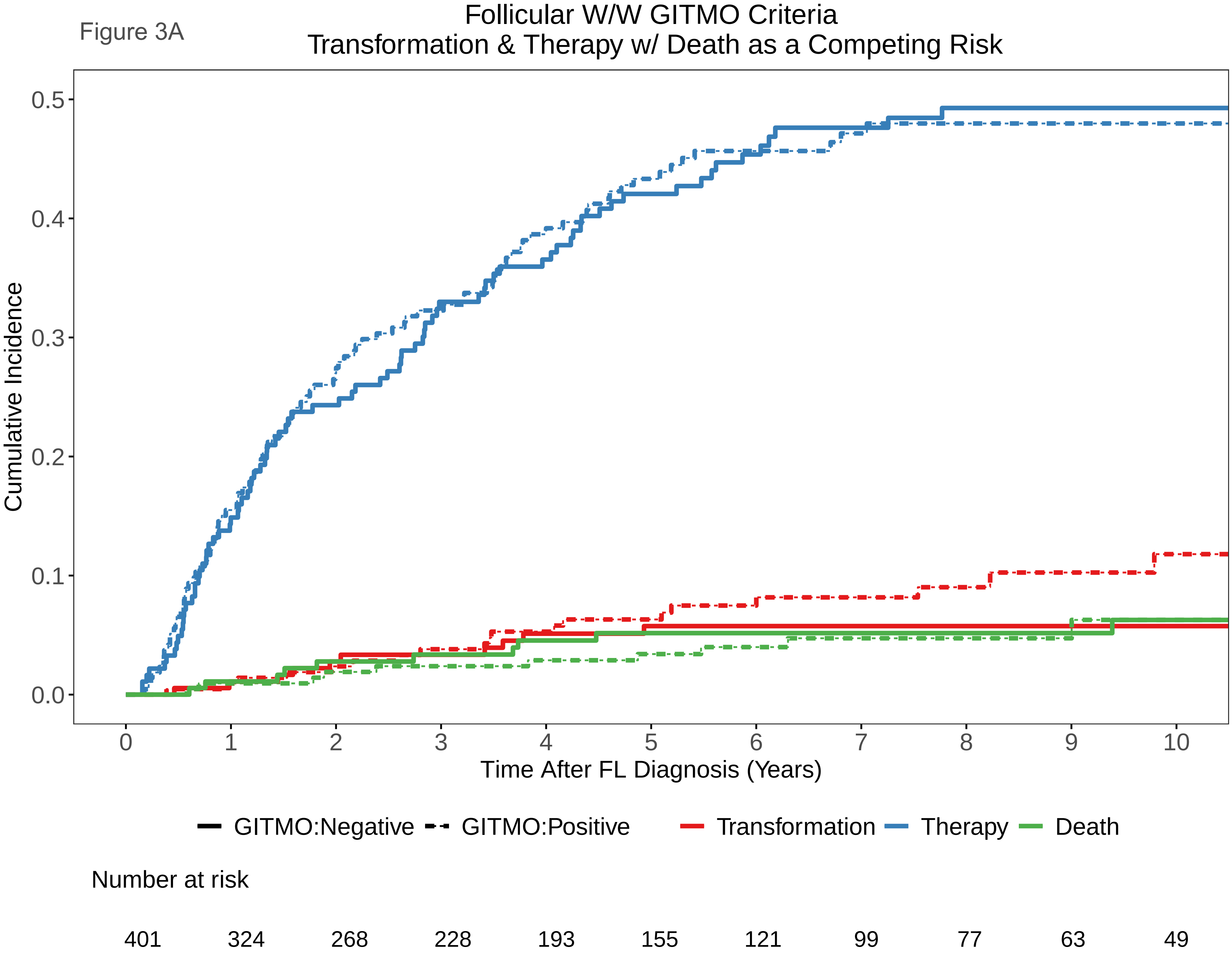

Follicular W/W GITMO Criteria
Transformation & Therapy w/ Death as a Competing Risk
Cumulative Incidence
0.0
0.1
0.2
0.3
0.4
0.5
0
1
2
3
4
5
6
7
8
9
10
Time After FL Diagnosis (Years)
GITMO:Negative
GITMO:Positive
Transformation
Therapy
Death
Number at risk
401
324
268
228
193
155
121
99
77
63
49

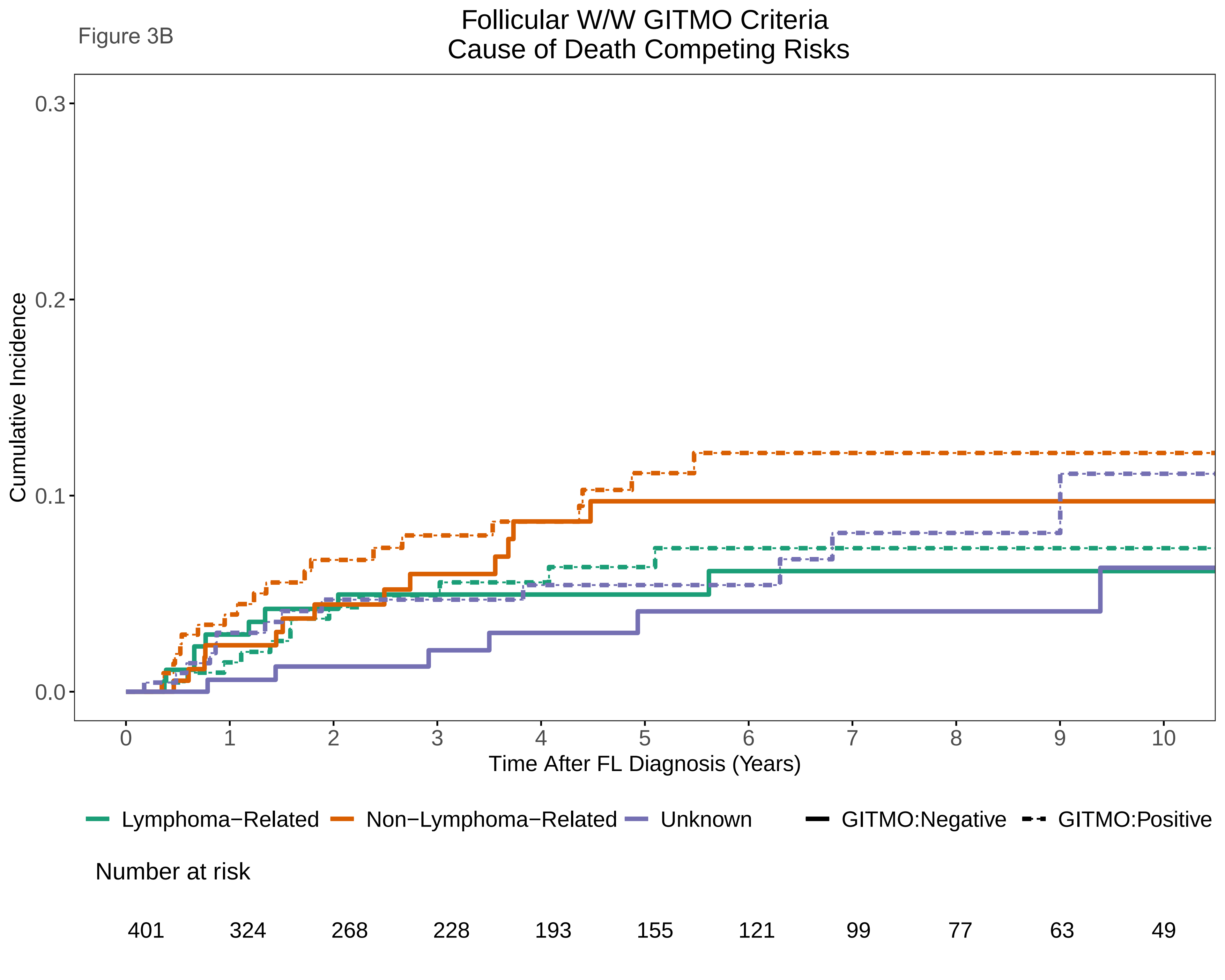

Figure 3B
Follicular W/W GITMO Criteria
Cause of Death Competing Risks
Cumulative Incidence
0.0
0.1
0.2
0.3
0
1
2
3
4
5
6
7
8
9
10
Time After FL Diagnosis (Years)
Lymphoma−Related
Non−Lymphoma−Related
Unknown
GITMO:Negative
GITMO:Positive
Number at risk
401
324
268
228
193
155
121
99
77
63
49
